# Polyomavirus Infection in Two Young Parrots: Assessment of Viral Distribution in Organs and Affected Tissues

**DOI:** 10.1155/vmi/2135685

**Published:** 2026-08-23

**Authors:** Gaia Casalino, Davide Messina, Clara Tolini, Giancarlo Bozzo, Antonio Lavazza, Francesco D’Amico, Antonella Bove, Francesco Pellegrini, Francesco D’Onghia, Diana Romito, Vito Martella, Canio Buonavoglia, Antonio Camarda, Elena Circella

**Affiliations:** ^1^ Department of Veterinary Medicine, University of Bari, S.P. Casamassima km. 3 70010 Valenzano, Bari, Italy, uniba.it; ^2^ Division of Veterinary Clinical Science, School of Veterinary Medicine and Science, Faculty of Medicine and Health Sciences, University of Nottingham, Sutton Bonington Campus, Loughborough LE12 5RD, UK, nottingham.ac.uk; ^3^ Reparto Virologia, Istituto Zooprofilattico Sperimentale Della Lombardia e Dell’Emilia-Romagna, Via Antonio Bianchi 7/9, 25124, Brescia, Italy, izsler.it; ^4^ Azienda Sanitaria Locale di Alessandria, Servizio Veterinario, Distretto di Tortona, Corso Romita 25/B, Tortona 15057, Italy; ^5^ Department of Pharmacology and Toxicology, University of Veterinary Medicine, Budapest 1078, Hungary, univet.hu

**Keywords:** APyV, avian polyomavirus, electron microscopy, genome sequencing, inclusion bodies, parrots, real-time PCR

## Abstract

Avian polyomavirus (APyV) is responsible for diseases in birds belonging to several avian orders, including Psittaciformes, Anseriformes, and other susceptible taxa. APyVs appear to be able to replicate in various organs of susceptible bird species. This study aimed to investigate (i) the distribution of APyV in the organs of two parrot chicks belonging to different species, Moluccan eclectus (*Eclectus roratus*) and blue‐winged macaw (*Primolius maracana*), which died due to APyV infection and (ii) the lesions associated with the infection. By negative‐staining electron microscopy, well‐defined viral particles morphologically compatible with polyomavirus and polyomavirus‐like particles were identified in organs. Detection of APyV was confirmed by real‐time PCR, and BLAST analysis of the complete genome revealed 99.96% identity with a budgerigar fledging disease polyomavirus strain. The amount of DNA copy number/ng of DNA ranged from 2.47 × 10^3^ found in the myocardium to 3.90 × 10^6^ found in the feather quills of the eclectus and from 1.07 × 10^1^ to 1.82 × 10^7^ detected in the brain and intestine of the blue‐winged macaw, respectively. According to the findings of inclusion bodies, viral particles, and DNA, the lung appeared as one of the most severely affected tissues. Even if inclusion bodies were not detected, severe lesions were found in the myocardium, bursa of Fabricius, and spleen. The results of this study highlight the ability of APyV to spread widely, leading to severe lesions in the organs of infected birds. The most significant amount of viral DNA detected in the gut and feathers highlights the relevance of feces and feathers as useful samples for the in vivo diagnosis.

## 1. Introduction

Polyomaviruses are non‐enveloped, icosahedral viruses with a double‐stranded, circular DNA of approximately 5000 bp and 5 to 6 open reading frames. The family Polyomaviridae is divided into six genera: *Alphapolyomavirus, Betapolyomavirus, Gammapolyomavirus, Deltapolyomavirus, Epsilonpolyomavirus,* and *Zetapolyomavirus* [1–3]. *Polyomavirus* genera infect different hosts with some specificity: alpha‐, beta‐, and delta‐*polyomaviruses* infect humans and other mammals mainly, while gamma‐, epsilon‐, and zeta‐*polyomaviruses* infect birds, whales, and dolphins, respectively. Polyomavirus‐like sequences have been also detected in several fish species [3, 4].

Mammalian polyomaviruses (MPyVs) are widely distributed among humans and other mammals, typically establishing subclinical or asymptomatic infections in their natural hosts, with diseases occurring primarily in immunocompromised individuals [5–8].

Avian Polyomaviruses (APyVs) exhibit unique biological characteristics, including a broad host range, higher pathogenicity, and organ tropism, compared to MPyVs [9]. Ten different APyVs are included in the genus *Gammapolyomaviruses*: Adélie penguin polyomavirus (AdPyV), budgerigar fledgling disease virus (BuFDV), butcherbird polyomavirus (Butcherbird PyV), canary polyomavirus (CaPyV), cormorant polyomavirus (CoPyV), crow polyomavirus (CPyV), *Erythrura gouldiae* polyomavirus (EgouPyV), finch polyomavirus (FPyV), goose hemorrhagic polyomavirus (GHPyV), and Hungarian finch polyomavirus (HunFPyV) [3, 10–14].

Unlike MPyVs, which typically cause disease in cases of immunodeficiency, APyVs cause disease even in fully immunocompetent birds [15]. APyV infections have been reported in a wide range of avian species belonging to the orders Psittaciformes, Passeriformes, and Galliformes other than in pigeons. Host susceptibility depends on the species and immune status, and on factors such as stress, animal flock density, and co‐infections that can significantly influence the course of infection [3, 16, 17]. The species belonging to the Order Psittaciformes are the most sensitive. Among these, budgerigars (*Melopsittacus undulatus*), lovebirds (*Agapornis* spp.), cockatiels (*Nymphicus hollandicus*) and macaws (*Ara* spp.) show high susceptibility, with severe clinical signs and high mortality rates, especially in nestlings and young birds. Adults often act as asymptomatic carriers [10, 18–22]. *Budgerigar fledgling disease polyomavirus,* which had been first identified in budgerigars in 1981 and has been also detected in other parrots worldwide [21–39], causes hepatitis, ascites, pericardial effusion, and abdominal distension [30, 31]. Chronically infected parrots often exhibit abnormal feather growth [32]. In addition, some budgerigars become persistently infected [30, 31]. Natural infections have been described in ornamental species such as canaries (*Serinus canaria*), finches, pigeons (*Columba livia*), and domestic poultry, generally with milder clinical manifestations compared to those observed in psittacines [18, 35]. However, CaPyV and FPyV can be responsible for mortality and lesions such as subcutaneous hemorrhages, enlargement of spleen and liver, extensive degeneration and hemorrhages in the center of the hepatic lobules, depletion of the spleen, and intranuclear inclusion bodies in liver, in spleen, and in the epithelium of renal glomeruli [36, 37]. Also, GHPyV causes ascites, subcutaneous edema, renal enlargement, and hemorrhagic nephritis and enteritis, other than lymphoid depletion in the Bursa of Fabricius, in young geese [38]. APyV appears to be able to replicate in multiple organs of various avian hosts [37–39]. Following viremia, the virus spreads throughout the host reaching multiple organs where it replicates and is subsequently excreted through various routes, including skin scaling, saliva, feces, and urine [37, 39–42]. The virus is eliminated in the feces for a few months after the infection has occurred [43]. These infected biological materials represent an important source of environmental contamination due to the significant environmental resistance of the virus, which can persist for long periods and be easily transmitted to susceptible hosts [3]. Furthermore, vertical transmission of APyV has been reported in geese [44] and, more recently, in finches [45]. In this latest study, virus detection in eggs was associated with both early and late embryonic mortality [45]. The aim of this study was to analyze the distribution and concentration of APyV in the organs of naturally infected blue‐winged macaw and Moluccan eclectus using real‐time PCR and to assess the possible relationship between viral load and lesions.

## 2. Material and Methods

### 2.1. Animals

The study was carried out on two parrots belonging to different species: a male Moluccan eclectus (*Eclectus roratus*) and a male blue‐winged macaw (*Primolius maracana*)*.* They came from a store where, usually, about two hundred baby parrots from different Italian farms were collected to complete their weaning by hand‐rearing and then resold to shops and individual buyers. A loss of weight was observed in the blue‐winged macaw. It was treated with clavulanate acid/amoxicillin (for a week) and nystatin (for 15 days) in the store, but unfortunately, it died after 15 days from the beginning of the treatment when it was 1.5 months old. The eclectus was sold when it was 2 months old. It quickly appeared lethargic and died 3 days after it was bought. The store manager did not provide any information concerning the clinical conditions of the other parrots. Both parrots were sent for necropsy to the Avian Diseases unit of the Department of Veterinary Medicine, University of Bari, Italy.

### 2.2. Bacteriology

Samples of liver and blood from the heart of both parrots were tested on blood agar (Triptic Soy agar, Basingstoke, UK, supplemented with 5% sheep blood) and McConkey agar (OXOID, Basingstoke, UK) and incubated at 37°C for 24 h. In addition, based on the gross lesions found in the eclectus, samples of air sacs were tested on the same media, and material from granulomas was plated on Sabouraud dextrose agar (Oxoid, Basingstoke, UK).

### 2.3. Histopathology

Samples of liver, lung, kidney, bursa of Fabricius, spleen, and heart from both parrots were collected for histological examination. Briefly, the tissue blocks were fixed in 10% neutral buffered formalin. The samples were embedded in paraffin wax, sectioned at 4 μm, and stained with hematoxylin and eosin (H&E).

### 2.4. Electron Microscopy (EM)

Samples of spleen, lung, liver, kidney, gizzard, small intestine, and feather quills were cut and mechanically homogenized in distilled water (ratio 1:5 w/v) for negative‐staining EM examination. The method [46] is based on the concentration of viral particles through ultracentrifugation for 15 min at 20psi with Airfuge (Beckman Coulter Inc., Brea, CA), mounting an A100 fixed‐angle rotor with 150 mL microtubes carrying specific adapters that allow the direct pelleting of the particle elements contained in the sample under examination onto 3 mm carbon‐treated copper grids covered with formvar film. Grids were stained with 2% sodium phosphotungstic (NaPT), pH 6.8, for 1.5 min and examined at 19–30000 with a Tecnai G2 Spirit transmission electron microscope (TEM) (Thermo Fisher Scientific, Eindhoven, The Netherlands) operating at 85 kV. Viral particles were identified based on their morphological characteristics.

### 2.5. Preliminary Screening for Circovirus and Polyomavirus by Polymerase Chain Reaction (PCR)

Samples of liver, spleen, and kidney from both birds were preliminarily screened by PCR for *circovirus* [47] and *polyomavirus* [37] using protocols previously described. All the samples were negative for *circovirus* but positive for *polyomavirus.* Therefore, aiming to investigate polyomavirus infection more thoroughly, we decided to test different tissues.

### 2.6. Polyomavirus Genome Amplification and Phylogenetic Analysis

The complete genome of viruses detected from liver samples from each parrot was amplified using the TempliPhi 100 kit (Cytiva, Hopkinton, MA 01748, USA) following a rolling circle amplification (RCA) protocol as described by Rector et al. (2004) [48]. Briefly, 1 μL of DNA was combined with 5 μL of TempliPhi sample buffer containing 450 mM dNTPs. The mixture was heated to 95°C for 3 min and then immediately cooled on ice. Subsequently, 5 μL of TempliPhi reaction buffer and 0.2 μL of TempliPhi enzyme mix were added and the reaction was incubated at 30°C for 16 h, followed by enzyme inactivation at 65°C for 10 min. For cloning purposes, 30 μL of the RCA product was digested with *Apa*I and the resulting ∼2 kb fragment was ligated into the pBluescript II SK(+) vector digested with *Apa*I (Stratagene, La Jolla, CA, USA) and introduced into *Escherichia coli* XL1‐Blue MRF’ cells (Stratagene, La Jolla, CA, USA). The 2 kb insert was sequenced using forward and reverse M13 universal primers (Invitrogen) at BaseClear BV. (Leiden, Netherlands). Consensus sequences were generated from three independent clones. Genomes were assembled and analyzed using the Geneious software suite (https://www.geneious.com), as well as tools available via NCBI (https://www.ncbi.nlm.nih.gov) and EMBL (https://www.ebi.ac.uk). The sequences from the Moluccan eclectus and the blue‐winged macaw were deposited in GenBank under accession numbers PZ157466 (BR1PRIMOLIUS_MARACANA_GA) and PZ157467 (BR2_ECLECTUS_RORATUS‐ELE). Phylogenetic analyses were performed with MEGA 4.1 Beta [48], constructing genome‐wide phylogenetic trees with both neighbor‐joining and parsimony approaches, and node support was assessed using > 1000 bootstrap replicates. To assess similarity to other polyomaviruses, 19 complete genomic sequences of APyVs were selected as listed in Table 1. The sequences were aligned and used to build the phylogenetic tree using the CLC Main Workbench 25 software.

**TABLE 1:** APV complete genome (PZ157467): nucleotide identities (%) with reference strains.

Virus name	Accession number	Sequence length (bp)	Host	Geographic origin	Collection year	Complete genome identity (%)
Budgerigar fledgling disease polyomavirus isolate PL1068X	KT203767.1	4981	*Melopsittacus undulatus*	Poland	2015	99.96
Gammapolyomavirus avis strain 19AD045	OQ230286.1	4981	*African grey parrot*	South Korea	2023	99.70
Gammapolyomavirus avis isolate CBNU_APyV_6	OM640122.1	4981	*Pionites melanocephalus*	South Korea	2022	99.68
Budgerigar fledgling disease polyomavirus DNA, strain: APV6	AB453164.1	4981	*Pionites melanocephala*	Japan	2009	99.60
Budgerigar fledgling disease polyomavirus	AY672646.1	4981	*Melopsittacus undulatus*	China	2008	99.56
Gammapolyomavirus avis isolate PT25528	KX008968.1	4981	*Ara chloroptera*	Portugal	2018	99.48
Budgerigar fledgling disease polyomavirus isolate PL1025B	KT203764.1	4981	*Psittacula krameri*	Poland	2015	99.46
Budgerigar fledgling polyomavirus	AF118150.1	4981	*Pteroglossus viridis*	Texas	1999	99.44
Gammapolyomavirus avis isolate LBF_41	OR004157.1	4981	*Agapornis roseicollis*	Arizona	2023	99.42
Aves polyomavirus 1 strain Taiwan‐12	MN123783.1	4980	*Guaruba guarouba*	Taiwan	2019	99.42
Avian polyomavirus strain APV‐P	MK061528.1	4981	*Columba livia*	China	2018	99.40
Gammapolyomavirus avis isolate LBF_56	OR004161.1	4981	*Agapornis roseicollis*	Arizona	2023	99.34
Aves polyomavirus 1 strain Taiwan‐17	MN123786.1	4982	*Vini kuhlii*	Taiwan	2019	99.34
Budgerigar fledgling disease polyomavirus isolate PL1233X	KT203769.1	4981	*Psittacula krameri*	Poland	2015	99.32
Gammapolyomavirus avis isolate BFPyV HB	PV506317.1	4981	*Nymphicus hollandicus*	China	2025	99.28
Budgerigar fledgling disease polyomavirus	FJ385773.1	4981	*Melopsittacus undulatus.*	China	2008	99.24
Aves polyomavirus 1 strain Taiwan‐3	MN047453.1	4971	*Pyrrhura picta*	Taiwan	2019	99.20
Gammapolyomavirus avis isolate LBF_42	OR004158.1	4981	*Agapornis roseicollis*	Arizona	2025	99.10
Gammapolyomavirus avis isolate SC‐YB19	MT119153.1	4963	*Melopsittacus undulatus*	China	2020	99.04

### 2.7. Polyomavirus Investigation by Real‐Time PCR

Twelve organs (brain, bursa of Fabricius, eye, feathers, gut, kidney, liver, lung, myocardium, proventriculus, spleen, and testicle) from the blue‐winged macaw and 16 organs (air sac, brain, bursa of Fabricius, eye, feathers, gizzard, gut, Harderian gland, kidney, liver, lung, myocardium, esophagus, proventriculus, spleen, and testicle) from the Moluccan eclectus were included in the study. The quantification of APyV DNA copies was performed by a real‐time PCR (qPCR). Amplification reactions were carried out using the SsoAdvanced Universal SYBR Green Supermix (BioRad, Milan, Italy) in a total reaction volume of 20 μL, comprising 18 μL of reaction mix with primers APyVgenf (CTDGATGARAATGGGGTKGG) and APyVgenr (ACMCGWACYTCCTCMACCT), at a final concentration of 5 pmol/μL, and 2 μL of template DNA. All reactions were conducted in triplicate. The qPCR assays were executed on QuantStudio 6 systems using SkanIt Software 7.01 RE (Applied Biosystems, Milan, Italy) and on the Bio‐Rad CFX Maestro Connect platform. The thermal cycling protocol consisted of an initial denaturation step at 98°C for 3 min, followed by 39 cycles of denaturation at 98°C for 15 s and annealing at 60°C for 45 s. Melting curve analysis was performed by increasing the temperature from 65°C to 95°C in 0.5°C increments every 5 s. Subsequently, a recombinant plasmid containing the target amplicon, propagated in the Escherichia coli MACH1 strain, was used as the quantitative standard. Plasmid DNA was extracted using the PureLink Quick Plasmid Miniprep Kit (Thermo Fisher Scientific, Petaluma, CA, USA), and its concentration was measured by UV spectrophotometry. The number of DNA copies was quantified according to the size of the plasmid and then converted to a copy number. A standard curve was generated using four 10‐fold serial dilutions of the plasmid, each tested in triplicate. Baseline settings and threshold cycle (Ct) values were automatically determined by the qPCR instrument software. For each positive sample, the mean viral DNA amount was normalized with respect to the concentration of the corresponding DNA extract.

### 2.8. Statistical Analysis

Descriptive statistics were used for data analysis. The figures derived from the qPCR test were introduced in Microsoft Excel for Microsoft 365 MSO (Version 2412 Build 16.0.18324.20092) 64 bit and displayed in bar and column charts.

## 3. Results

### 3.1. Gross Findings

**FIGURE 1:**
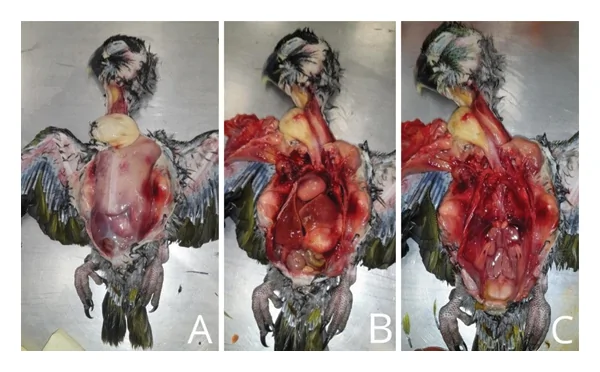
Prevalent macroscopic lesions of the blue‐winged macaw: (A) hemorrhages on the skin, subcutaneous tissue, and muscles. (B) Pallor of myocardium and degeneration of liver. (C) Pulmonary congestion and kidney pallor.

**FIGURE 2:**
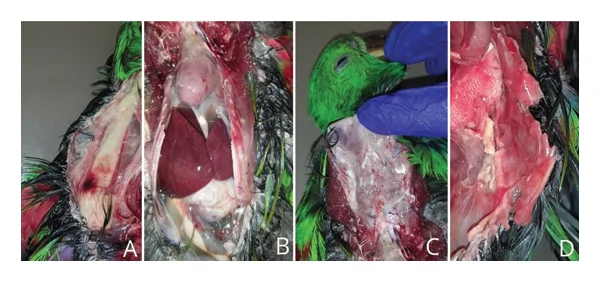
Prevalent macroscopic lesions of the Moluccan eclectus: (A) Large hemorrhage on the subcutaneous tissue and external surface of the esophagus. (B) Hydropericardium, hemorrhages on the pericardium, hepatitis with degeneration areas, and white and round granuloma on the pericardium. (C) Caseous exudate and hemorrhages on the inner surface of the sternal keel. (D) Caseous exudate in the thoracic air sac.

At necropsy, the most prevalent lesions found in the blue‐winged macaw were the hemorrhages, which were observed on the skin and were particularly widespread in the subcutaneous tissue and on the muscles (Figure 1A). Pallor of myocardium, enlargement and discoloration of liver (Figure 1B), and kidneys (Figure 1C) were also detected. Some hemorrhages (Figure 2A) were also observed in the subcutaneous tissue of the eclectus. However, hydropericardium, hemorrhages on the pericardium, small hemorrhages and altered areas on the liver surface (Figure 2B), hypoplasia of the spleen, and discoloration of the kidneys were the gross lesions found in the eclectus. Caseous exudate was also found on the inner surface of the sternal keel (Figure 2C) and in the air sacs (Figure 2D). In addition, granulomas compatible with aspergillosis were found on the pericardium (Figure 2B) and the thoracic air sac.

### 3.2. Bacteriology

**FIGURE 3:**
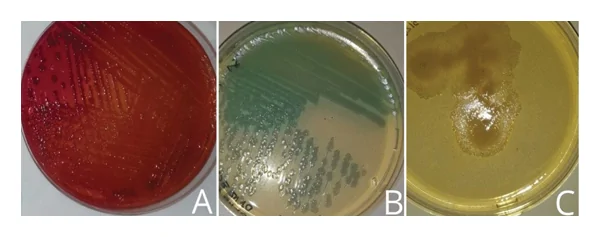
Secondary infections. Blue‐winged macaw: (A) *Escherichia coli* from liver of on Mc Conkey agar. Moluccan eclectus: (B) *Pseudomonas aeruginosa* from hearth blood on TSA. (C) *Aspergillus* spp. from the air sac on Sabouraud agar.

*Escherichia coli* was isolated from the liver and the heart of the blue‐winged macaw (Figure 3A). In contrast, *Pseudomonas aeruginosa* (Figure 3B) was isolated from all the tested tissues of the eclectus and *Aspergillus* spp. (Figure 3C) was isolated from the granuloma material.

### 3.3. Histopathology

**FIGURE 4:**
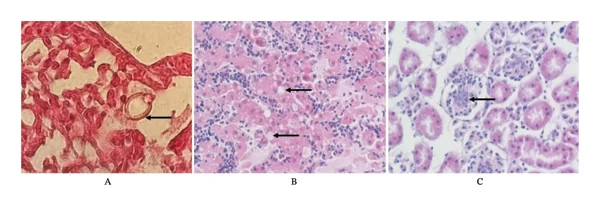
Hematoxylin and eosin stain: Cowdry type B intranuclear inclusion bodies in lung (100x) (A) and kidney (40x) (C) of the blue‐winged macaw and liver (40x) (B) of the eclectus.

Intranuclear Cowdry B‐type inclusions were detected mainly in liver sections, but the lung appeared as the most affected tissue in both birds. In detail, hemorrhagic areas, inflammatory cells, and total breakdown of the lung structure were found. The mucosa of the airways was acutely inflamed and congested, and lobular bronchi became filled with exudate composed of protein‐rich fluid and numerous heterophils, with strips of necrotic epithelium. Balloon‐shaped Cowdry type B intranuclear inclusion bodies with margination of basophilic chromatin were detected (Figure 4A)**.** Several intranuclear Cowdry type B bodies with margination of basophilic chromatin were found in the liver of both parrots (Figure 4B). The liver damage observed macroscopically in the eclectus was confirmed by histological findings, which revealed nodular inflammatory cells, hemorrhagic areas, and necrosis. In contrast, the lesions in the liver section of the blue‐winged macaw were less pronounced. Renal damage was evident in both birds. Epithelial cells of the proximal tubules were enlarged, with vacuolated cytoplasm. Degeneration of the epithelial cells in the whole cross‐section of the proximal tubules suggested tubulonephrosis. In addition, vacuolated cells and glomerular atrophy were observed. Cowdry B intranuclear inclusion bodies with margination of basophilic chromatin were found in the renal glomerulus (Figure 4C).

In both parrots, the histological sections of the bursa of Fabricius and spleen showed hemorrhagic areas and lymphoid depletion in the follicles. However, no Cowdry type B intranuclear inclusion bodies were found. Likewise, no Cowdry B intranuclear inclusion bodies were detected in the myocardial samples, but lesions were found in the samples from both parrots. Longitudinally oriented myocytes were thoroughly infiltrated centrally by lymphocytes, leading to tissue destruction. Additionally, the inflammatory infiltrate in the myocardium alternated between necrosis and degeneration. The severe inflammatory infiltrates throughout all myocardial samples were associated with edema and extensive myocyte damage.

### 3.4. Electron Microscopy

**FIGURE 5:**
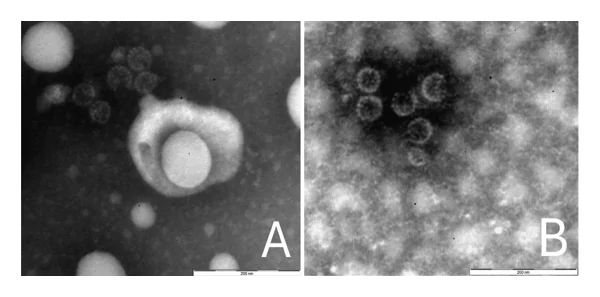
Ultramicrograph of virions morphologically resembling polyomavirus observed: (A) in the spleen extract from the blue‐winged macaw; (B) in the gut extract from the Moluccan eclectus. Negative staining 2% NaPT, pH 6.8. TEM FEI Tecnai G2 Spirit operating at 85 kV. Bar = 200 nm.

Icosahedral non‐enveloped viral particles of 40–45 nm in size, morphologically related to polyomavirus, were identified in various tissues (Table 2). They were found mainly in the spleen (Figure 5A) extracts of both parrots, in the lung and proventriculus of the blue‐winged macaw, and the gut (Figure 5B) and feathers of the Moluccan eclectus. In addition, polyomavirus‐like particles were identified in the tissue from the blue‐winged macaw as well as in lung and feather quill extracts from both parrots.

**TABLE 2:** Viral DNA detected by real‐time PCR and viral particles found by electron microscopy (EM) in the different tissues of the two parrots.

Tissue	Blue‐winged macaw		Moluccan eclectus	
	DNA copy number/ng	EM	DNA copy number/ng	EM
Air sac	NA	NA	4.13 × 10^4^	NA
Brain	1.07 × 10^1^	NA	2.70 × 10^4^	NA
Bursa of Fabricius	1.16 × 10^3^	NA	3.14 × 10^3^	NA
Eye	1.65 × 10^2^	NA	7.34 × 10^3^	NA
Feathers	2.41 × 10^4^	**+**	3.90 × 10^6^	**++**
Gizzard	NA	NA	2.27 × 10^4^	ND
Gut	1.82 × 10^7^	ND	2.84 × 10^4^	**++**
Harderian gland	NA	NA	3.95 × 10^3^	NA
Kidney	1.36 × 10^5^	ND	3.78 × 10^4^	NA
Liver	1.63 × 10^4^	NA	8.91 × 10^3^	ND
Lung	4.27 × 10^3^	**++**	5.38 × 10^5^	**+**
Myocardium	1.09 × 10^1^	NA	2.47 × 10^3^	NA
Esophagus	NA	NA	6.53 × 10^3^	NA
Proventriculus	2.33 × 10^6^	**++**	1.14 × 10^4^	ND
Spleen	8.02 × 10^5^	**++**	3.29 × 10^5^	**++**
Testicle	1.18 × 10^3^	NA	6.24 × 10^4^	NA

*Note:* ND: No viral particles detected; +: 1–5 viral particles detected across all observed grid meshes; ++: 1–5 viral particles detected in each examined mesh unit of the grid.

Abbreviation: NA, not analyzed.

### 3.5. Complete Genome Analysis

**FIGURE 6:**
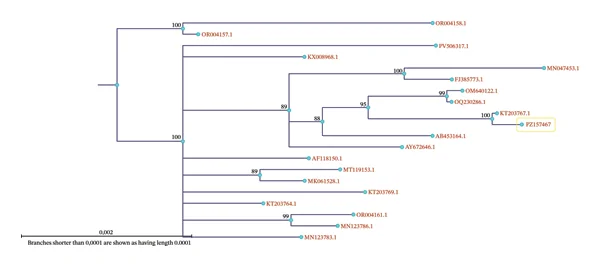
Phylogenetic tree constructed using the Neighbor‐Joining method with > 1000 bootstrap replicates to estimate branch support. Bootstrap values are shown on the tree. Bootstrap threshold was set at 70%.

BLAST analysis of the nucleotide sequences of the complete genomes of the detected viruses (PZ157466 and PZ157467) revealed that they were identical to each other. Therefore, there was only one sequence (PZ157467) for the phylogenetic tree. Comparative analysis showed 99.96% identity with *Budgerigar fledgling disease polyomavirus isolate PL1068X* (KT203767.1) (Table 1 and Figure 6). The lowest identity (99.04%) was found with *Gammapolyomavirus avis isolate SC-YB19* (MT119153.1).

### 3.6. Detection of APyV DNA by Real‐Time PCR

The amount of APyV DNA copy number/ng of tissue, which was detected in the organs of the two parrots, is reported in Table 2.

**FIGURE 7:**
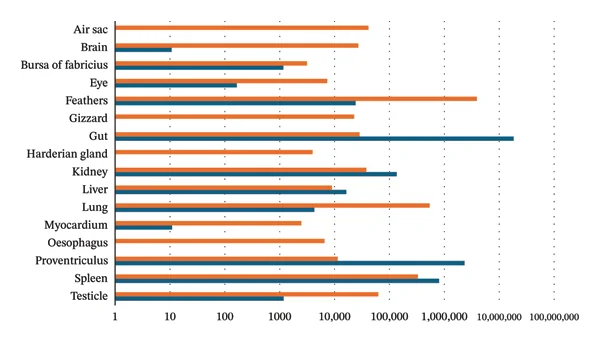
APV DNA (copy number/ng) distribution by organs and species: the eclectus in orange; the blue‐winged macaw in blue.

**FIGURE 8:**
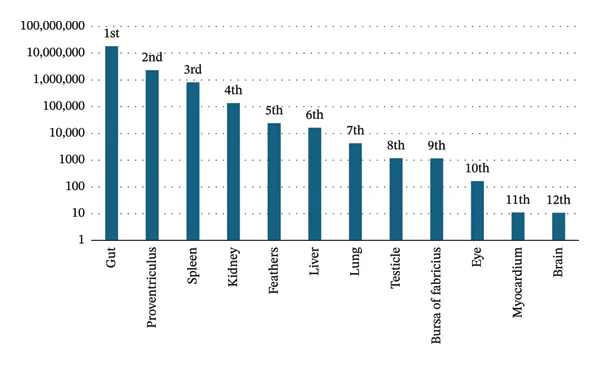
Organ rank by viral DNA detected in the blue‐winged macaw.

Some relevant differences in the viral DNA distribution in the organs of the two parrots were found (Figure 7). In detail, the gut was the most affected organ of the blue‐winged macaw, with 1.82 × 10^7^ copy number/ng of DNA detected in (Figure 8), while it was the seventh most affected organ of the eclectus with 2.84 × 10^4^ DNA copy number/ng.

**FIGURE 9:**
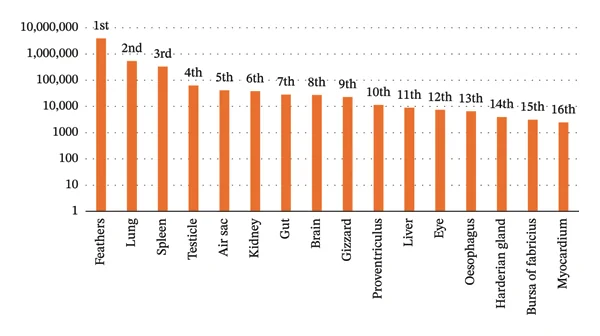
Organ rank by viral DNA detected in the eclectus.

The proventriculus, which was the second most affected organ of the blue‐winged macaw, had a 2.33 × 10^6^ DNA copy number/ng. In contrast, it had a 1.14 × 10^4^ DNA copy number/ng, making it the 10th most affected organ of the eclectus. By comparison, the feathers were the most affected tissue of the eclectus (Figure 9), having 3.90 × 10^6^ DNA copy number/ng. The feathers of the blue‐winged macaw had 2.41 × 10^4^ DNA copy number/ng, and they were the fifth most affected tissue in this species. Among the lymphoid tissues, the spleen was the third most affected organ in both parrots, with 8.02 × 10^5^ and 3.29 × 10^5^ DNA copy number/ng found in the blue‐winged macaw and the eclectus, respectively. Similar DNA copy number/ng were found in the bursa of Fabricius of the eclectus (3.14 × 10^3^) and blue‐winged macaw (1.16 × 10^3^). The lung appeared as the seventh most affected organ in the blue‐winged macaw, and it had 4.27 × 10^3^ DNA copy number/ng. Conversely, the number of DNA copies found in the lung of the eclectus was higher (5.38 × 10^5^), and this tissue appeared as the second most affected in this species.

## 4. Discussion

APyV infection was associated with the death of both tested parrots, which were less than 2 months old, confirming the high pathogenicity of APyV for nestling and young individuals [20, 49–51]. Both chicks came from the same center for weaning‐rearing. In this kind of store, large number of individuals also belonging to different species occurs, with several hatchlings encountering each other. These conditions can promote the spread of the virus, increasing the risk of occurrence of severe clinical forms with high mortality among highly susceptible individuals due to age and species, which are frequently observed in such nurseries [33, 52, 53]. Being very young, both baby parrots were probably very susceptible to the clinical infection because of their age and species. Accordingly, lesions involving several organs were found at necropsy. The gross lesions were predominantly found in the liver and kidneys, confirming the involvement of these tissues in the occurrence of APyV infection [54].

Analysis of the complete genome of the virus found in parrot organs confirmed the high level of genomic conservation of BR2_ECLECTUS_RORATUS‐ELE within APyVs. Furthermore, the high identity with a polyomavirus strain of parakeet disease bred in Poland suggests that an ancestral strain of APyV was likely responsible for the disease observed in parrot nestlings.

Regarding the distribution of the virus, it was quite different depending on the parrot. In fact, the amount of viral DNA was quite similar among the tissues of the eclectus, ranging from 2.47 × 10^3^ DNA copy number/ng found in the myocardium to 3.90 × 10^6^ DNA copy number/ng found in the feather quills. By contrast, in the blue‐wing macaw, the amount of viral DNA ranged from 1.07 × 10^1^ to 1.82 × 10^7^ copy number/ng, as detected in the brain and gut, respectively. Anyway, even if in different amounts according to the tissue and the parrot, viral DNA was detected in all tested samples, highlighting the broad range of tissues involved in the infection. This widespread tissue distribution agrees with the findings of Phalen et al. [39], although the two studies employed different approaches, with a semiquantitative PCR assay used by Phalen et al. and a quantitative real‐time PCR used in the present study. Despite some tissues being analyzed as pooled samples by Phalen et al., both studies demonstrated a broad distribution of viral DNA among organs. Feathers, lymphoid tissues, and the gastrointestinal tract were consistently involved in both studies. However, some differences in tissue distribution were observed, with the highest viral loads occurring in the gastrointestinal tract of the blue‐winged macaw, whereas feathers and lung tissue represented the most affected sites in the Moluccan eclectus. These findings suggest that, despite a common systemic pattern of infection, the relative distribution of APyV DNA may vary among host species and individuals. Furthermore, slight differences in viral distribution due to the age of the birds should not be excluded, considering the younger age of 1.5 and 2 months of the blue‐winged macaw and the eclectus, respectively, compared to 6 months of the budgerigars tested by Phalen et al. [39], age corresponding to sexual maturity in this species. The ability of APyVs to spread in the host without a specific tropism, unlike MPyVs, could be linked to several possible reasons. For example, some MPyVs such as JC polyomavirus, which is a human polyomavirus strain named after patient JC (John Cunningham) [55], seem to stimulate the expression of interferon‐α and interferon‐β in human cells with consequent decrease of their replication [56, 57]. By contrast, a study performed on *Budgerigar fledgling disease polyomavirus* highlighted that the proteins VP4 and VP4‐delta appear to be able to bind the promoter of interferon‐β, preventing the production of interferon‐β and leading APyVs to easily and massively replicate in the host cells [40]. VP4 is also considered implicated in the induction of apoptosis, which is typically caused by APyVs instead of necrosis, unlike MPyVs. Therefore, APyVs evade the immune response of the host more easily, spreading to the different organs of infected birds [3, 10, 18, 58]. In addition, APyVs seem to have tropism for endothelial cells that are widespread in all tissues, causing widespread hemorrhages [59], which can be frequently observed in the case of APyV infection. Accordingly, hemorrhages were found on the skin, subcutaneous tissue, and muscles of the blue‐winged macaw, and hemorrhages were also detected as histological lesions in the liver, lung, and bursa of Fabricius of both parrots. Other microscopic lesions had different severity depending on the organ involved. Tubular nephrosis, glomerular atrophy, and intranuclear inclusion bodies were observed in the kidneys of both parrots, where 1.36 × 10^5^ and 3.78 × 10^4^ APyV DNA copy number/ng were detected in the blue‐wing macaw and the eclectus, respectively. Although clinically evident glomerulopathies seem to be rarer in birds because they have a highly efficient glomerular filtration system, unlike mammals [54], severe microscopic lesions seem to be quite frequently found in birds affected by APyV infection. Accordingly, necrosis, hyperemia, infiltration of inflammatory cells [20, 54], and thickening of renal capillaries [52, 54] have been reported in psittacine species. Phalen et al. [52] found that the severity of glomerular lesions can differ among individuals in young non‐budgerigar parrots. Hemorrhagic [36] and interstitial nephritis [60] were observed in canaries and geese, respectively. In finches, APyV was responsible for tubular nephrosis‐necrosis with enlargement and degeneration of epithelial cells, thickening of the glomerular capillaries, and glomerular atrophy [37].

Inclusion bodies, chromatin margination, infiltration of inflammatory cells, hemorrhages, and necrosis were found in the liver, associated with 1.63 × 10^4^ and 8.91 × 10^3^ APyV DNA copy number/ng, which were found in the blue‐winged macaw and the eclectus, respectively. Similar lesions were found in nestlings of budgerigars infected at 8–19 days of age and that died within 16 months [61], in young birds belonging to different psittacine species described by Garcia et al. [62], and in an adult Amazon parrot (*Amazona autumnalis*), which was persistently infected [20]. Likewise, focal hepatitis with fibrosis, lymphocytic infiltration, areas of necrosis, and a high number of Cowdry type B intranuclear inclusion bodies in the hepatocytes were found in finches, confirming the massive involvement of the liver in APyV infection in birds belonging to the family *Fringillidae* [37]. The liver appears as one of the most affected organs in the canary (*Serinus canaria*), where extensive degeneration with hemorrhages and hemopoiesis was found in the liver of young individuals that died at about 40 days of age [36].

According to the amount of APyV DNA detected in the bursa of Fabricius and spleen tissues, hemorrhagic areas and lymphoid depletion in the follicles were found in both parrots. Those lesions were probably responsible for immunodepression that may have favored secondary infections due to *Escherichia coli* and *Pseudomonas aeruginosa* found in the blue‐winged macaw and eclectus, respectively. Severe lesions, including lymphoid depletion and necrosis, have been reported in various parrots [20, 54, 63]. Likewise, lymphoid depletion and virions were detected in the lymphoid tissues of geese infected with GHPyV [60]. A relationship between the sporadic or massive finding of viral particles and the detection of low or high amounts of viral DNA was hypothesized in a study performed on cockatiels and budgerigars [21]. Accordingly, viral particles, as single virions and organized in clusters, were found in the spleen of both the parrots, where more than 3 × 10^5^ copy number/ng were detected. Likewise, viral particles were detected mainly in some tissues such as the feathers of the eclectus and the proventriculus of the macaw, where the viral DNA resulted in more than 2 × 10^6^ copy number/ng. Conversely, viral particles were not detected in the gut of the macaw, where 1.82 × 10^7^ DNA copy number/ng was found. In addition, virions were not detected in tissues such as the liver of the eclectus and the kidney of the macaw where the DNA copy number was 8.91 × 10^3^ and 1.36 × 10^5^. Therefore, based on these results, the finding of viral particles in EM is not necessarily linked to the amount of viral DNA in the samples. Still, it could more likely be related to the non‐homogeneous and optimal state of preservation of the samples or dependent on other factors, such as the more or less acute stage of evolution of the disease at the time of sampling. Also, the amount of viral DNA, which was found in the tissues, was not always linked to the severity of lesions. For example, the lung appeared as one of the most affected tissues in both parrots. Similar severe lesions, such as hemorrhagic areas, inflammatory cells, congestion, necrosis, and inclusion bodies, were found. Still, they were associated with higher (5.38 × 10^5^) viral DNA copy number/ng detected in the lung of the Moluccan eclectus than in the lung of the blue‐winged macaw (4.27 × 10^3^). Likewise, despite the different amount of APyV DNA detected in myocardium (1.09 × 10^1^ in the blue‐winged macaw vs. 2.47 × 10^3^ in the eclectus), necrosis, degeneration, edema, and extensive myocyte damage were similarly found in both parrots.

## 5. Conclusions

To our knowledge, although the possible finding of APyV in several tissues has already been suggested for several years [64], this is the first study to investigate in depth the virus distribution in non‐budgerigar host on a case of natural infection. Even if in different amounts according to the tissue, viral DNA was detected in all tested samples, highlighting the ability of APyV to spread and replicate in a broad range of tissues. APyV was detected in large amounts in the liver, kidney, and lung. Still, one of the most interesting findings of this study, in our opinion, is that the highest amount of APyV DNA was detected in the gut sample of the blue‐wing macaw and in the feathers of the eclectus. Intestinal content was already suggested as a possible route of spreading of APyV because viral DNA was previously detected in the cloaca of both adults and young infected birds [20, 28, 49, 51, 65–67]. Our results, aside from highlighting the large number of viruses that can be potentially detected in feces, suggest that feathers are a relevant source of virus spread in the environment and may be important in the epidemiology of APyV infection. Therefore, feathers and feces, which appeared to be the most infected among the examined tissues, should be considered preferred samples, especially if collected and analyzed simultaneously, in in vivo diagnostic procedures.

## Funding

No funding was received for this manuscript. Open access publishing facilitated by Universita degli Studi di Bari Aldo Moro, as part of the Wiley ‐ CRUI‐CARE agreement.

## Ethics Statement

Ethical approval is not applicable to this study because it was conducted on the carcasses of parrots that had already died from a natural polyomavirus infection. The owners of deceased parrots were informed about the study objectives and gave their consent for their inclusion in the study.

## Conflicts of Interest

The authors declare no conflicts of interest.

## Data Availability

The data supporting this study are available from the corresponding author.
